# Immune checkpoint inhibitor therapy and outcomes from SARS-CoV-2 infection in patients with cancer: a joint analysis of OnCovid and ESMO-CoCARE registries

**DOI:** 10.1136/jitc-2022-005732

**Published:** 2022-11-30

**Authors:** Alessio Cortellini, Gino M Dettorre, Urania Dafni, Juan Aguilar-Company, Luis Castelo-Branco, Matteo Lambertini, Spyridon Gennatas, Vasileios Angelis, Ailsa Sita-Lumsden, Jacobo Rogado, Paolo Pedrazzoli, David Viñal, Aleix Prat, Maura Rossi, Rossana Berardi, Teresa Alonso-Gordoa, Salvatore Grisanti, Georgia Dimopoulou, Paola Queirolo, Sylvain Pradervand, Alexia Bertuzzi, Mark Bower, Dirk Arnold, Ramon Salazar, Marco Tucci, Kevin J Harrington, Francesca Mazzoni, Uma Mukherjee, Zoi Tsourti, Olivier Michielin, Fanny Pommeret, Joan Brunet, Bruno Vincenzi, Giuseppe Tonini, Andrea Patriarca, Federica Biello, Marco Krengli, Josep Tabernero, George Pentheroudakis, Alessandra Gennari, Solange Peters, Emanuela Romano, David J Pinato

**Affiliations:** 1 Department of Surgery & Cancer, Hammersmith Hospital Campus, Imperial College London, London, UK; 2 Medical Oncology, Fondazione Policlinico Universitario Campus Bio-Medico, Via Alvaro del Portillo, 200 - 00128, Roma, Italy; 3 Department of Internal Medicine, Washington University School of Medicine, St. Louis, Missouri, USA; 4 Laboratory of Biostatistics, School of Health Sciences, National and Kapodistrian University of Athens, Athens, Greece; 5 Medical Oncology, Vall d'Hebron University Hospital and Institute of Oncology (VHIO), Barcelona, Spain; 6 Infectious Disease, Vall d'Hebron University Hospital, Barcelona, Spain; 7 Scientific and Medical Division, ESMO (European Society for Medical Oncology), Lugano, Switzerland; 8 NOVA National School of Publich Health, NOVA University, Lisbon, Portugal; 9 Department of Internal Medicine and Medical Specialties (DiMI), School of Medicine, University of Genoa, Genova, Italy; 10 Medical Oncology Department, U.O. Clinica di Oncologia Medica, IRCCS Ospedale Policlinico San Martino, Genova, Italy; 11 Medical Oncology Department, The Royal Marsden Hospital and NHS Foundation Trust, London, UK; 12 Medical Oncology, Guy's and St Thomas' NHS Foundation Trust (GSTT), London, UK; 13 Medical Oncology Department, Hospital Universitario Infanta Leonor, Madrid, Spain; 14 Medical Oncology, Fondazione IRCCS Policlinico San Matteo, Pavia, Italy; 15 Department of Internal Medicine and Medical Therapy, University of Pavia, Pavia, Italy; 16 Medical Oncology Department, Hospital Universitario La Paz, Madrid, Spain; 17 Department of Medical Oncology, Hospital Clinic de Barcelona, Barcelona, Spain; 18 Translational Genomics and Targeted Therapies in Solid Tumors, Institut d'Investigacions Biomediques August Pi i Sunyer (IDIBAPS), Barcelona, Spain; 19 Oncology Department, ASO ‘SS Antonio Biagio e Cesare Arrigo’, Alessandria, Italy; 20 Medical Oncology, AOU Ospedali Riuniti, Polytechnic University of the Marche Region, Ancona, Italy; 21 Medical Oncology Department, Hospital Universitario Ramón y Cajal, Madrid, Spain; 22 Medical Oncology Unit, Spedali Civili, Brescia, Italy; 23 Melanoma Sarcoma and Rare Tumors, IEO, European Institute of Oncology IRCCS, Milan, Italy; 24 Oncology Department, Centre Hospitalier Universitaire Vaudois (CHUV), Lausanne, Switzerland; 25 Medical Oncology and Hematology Unit, Humanitas Cancer Center, IRCCS Humanitas Research Hospital, Rozzano, Milan, Italy; 26 Department of Oncology and National Centre for HIV Malignancy, Chelsea and Westminster Hospital, London, UK; 27 Oncology, Haematology, Palliative Care Department, Asklepios Klinik Altona e Asklepios Kliniken, Hamburg, Germany; 28 Department of Medical Oncology, ICO L’Hospitalet, Oncobell Program (IDIBELL), CIBERONC, Hospitalet de Llobregat, Barcelona, Spain; 29 Section of Medical Oncology, Department of Interdisciplinary Medicine (DIM), University of Bari 'Aldo Moro', Bari, Italy; 30 IRCCS Istituto Tumori Giovanni Paolo II, Bari, Italy; 31 Division of Radiotherapy and Imaging, The Royal Marsden Hospital and The Institute of Cancer Research NIHR Biomedical Research Centre, London, UK; 32 Medical Oncology, Careggi University Hospital, Florence, Italy; 33 Medical Oncology, Barts Health NHS Trust, London, UK; 34 Frontier Science Foundation-Hellas, Athens, Greece; 35 Department of Cancer Medicine, Institut Gustave Roussy, University of Paris Saclay, 114 rue Edouard Vaillant, Villejuif, France; 36 Department of Medical Oncology, Catalan Institute of Oncology, University Hospital Josep Trueta, Girona, Spain; 37 Division of Haematology, Department of Translational Medicine, University of Piemonte Orientale and Azienda Ospedaliera Maggiore della Carità, Novara, Italy; 38 Division of Oncology, Department of Translational Medicine, University of Piemonte Orientale and Azienda Ospedaliera Maggiore della Carità, Novara, Italy; 39 Division of Radiotherapy, Department of Translational Medicine, University of Piemonte Orientale and Azienda Ospedaliera Maggiore Della Carita, Novara, Italy; 40 Medical Oncology, Vall d'Hebron University Hospital and Institute of Oncology (VHIO), IOB-Quiron, UVic-UCC, Barcelona, Spain; 41 Center for Cancer Immunotherapy, Department of Oncology, PSL Research University, Institut Curie, Paris, France

**Keywords:** COVID-19, Vaccination, Immunogenicity, Vaccine, Cytotoxicity, Immunologic, Immunotherapy

## Abstract

**Background:**

As management and prevention strategies against COVID-19 evolve, it is still uncertain whether prior exposure to immune checkpoint inhibitors (ICIs) affects COVID-19 severity in patients with cancer.

**Methods:**

In a joint analysis of ICI recipients from OnCovid (NCT04393974) and European Society for Medical Oncology (ESMO) CoCARE registries, we assessed severity and mortality from SARS-CoV-2 in vaccinated and unvaccinated patients with cancer and explored whether prior immune-related adverse events (irAEs) influenced outcome from COVID-19.

**Findings:**

The study population consisted of 240 patients diagnosed with COVID-19 between January 2020 and February 2022 exposed to ICI within 3 months prior to COVID-19 diagnosis, with a 30-day case fatality rate (CFR_30_) of 23.6% (95% CI 17.8 to 30.7%). Overall, 42 (17.5%) were fully vaccinated prior to COVID-19 and experienced decreased CFR_30_ (4.8% vs 28.1%, p=0.0009), hospitalization rate (27.5% vs 63.2%, p<0.0001), requirement of oxygen therapy (15.8% vs 41.5%, p=0.0030), COVID-19 complication rate (11.9% vs 34.6%, p=0.0040), with a reduced need for COVID-19-specific therapy (26.3% vs 57.9%, p=0.0004) compared with unvaccinated patients. Inverse probability of treatment weighting (IPTW)-fitted multivariable analysis, following a clustered-robust correction for the data source (OnCovid vs ESMO CoCARE), confirmed that vaccinated patients experienced a decreased risk of death at 30 days (adjusted OR, aOR 0.08, 95% CI 0.01 to 0.69).

Overall, 38 patients (15.8%) experienced at least one irAE of any grade at any time prior to COVID-19, at a median time of 3.2 months (range 0.13–48.7) from COVID-19 diagnosis. IrAEs occurred independently of baseline characteristics except for primary tumor (p=0.0373) and were associated with a significantly decreased CFR_30_ (10.8% vs 26.0%, p=0.0462) additionally confirmed by the IPTW-fitted multivariable analysis (aOR 0.47, 95% CI 0.33 to 0.67). Patients who experienced irAEs also presented a higher median absolute lymphocyte count at COVID-19 (1.4 vs 0.8 10^9^ cells/L, p=0.0098).

**Conclusion:**

Anti-SARS-CoV-2 vaccination reduces morbidity and mortality from COVID-19 in ICI recipients. History of irAEs might identify patients with pre-existing protection from COVID-19, warranting further investigation of adaptive immune determinants of protection from SARS-CoV-2.

**WHAT IS ALREADY KNOWN ON THIS TOPIC**

- SARS-CoV-2 vaccines significantly improve COVID-19 morbidity and mortality in patients with cancer. Efficacy data from large registry studies in patients receiving immune checkpoint inhibitors (ICIs) are still lacking.

**WHAT THIS STUDY ADDS**

- This joint analysis of patients recently exposed to ICI from OnCovid and European Society for Medical Oncology-CoCARE registries confirms clinical efficacy of SARS-CoV-2 vaccination in reducing COVID-19 morbidity and mortality.

**HOW THIS STUDY MIGHT AFFECT RESEARCH, PRACTICE OR POLICY**

- Considering the continuously expanding indication for ICI therapy, these findings are of the utmost importance to ensure effective utilization of this therapy during and beyond the SARS-CoV-2 global pandemic.

## Introduction

The efficacy of immune checkpoint inhibitors (ICIs) strongly relies on their capacity of inducing T-cell immune reconstitution.^1^ T-cell exhaustion is a contributory mechanism underlying the severity of SARS-CoV-2 infection,^2^ leading on one hand to the investigation of programmed-cell death-1 inhibitors as a therapeutic strategy in severe COVID-19.^3^ On the other hand, given the pathological immune-mediated mechanisms underlying COVID-19 and the risk of immune-pathology stemming from ICI use, there has been growing concern around the use of ICI in patients with COVID-19 and cancer.^4 5^

Clinical data in support of a protective, as opposed to detrimental, effect of ICI in the prognosis of COVID-19 in patients with cancer have been inevitably biased by patient selection and underlying clinical characteristics. Initial reports revealed inconsistent results ranging from worse outcomes,^6 7^ to no difference in COVID-19 severity^8 9^ in ICI-exposed patients compared with ICI-unexposed patients.

Large metanalyses have suggested no differential impact of ICIs on COVID-19 morbidity and mortality in comparison to other systemic anticancer therapies.^10 11^

However, COVID-19 outcomes in patients with cancer have substantially evolved over time. Improved management of COVID-19,^12^ immunization campaigns,^13 14^ changes in community transmission and the emergence of new SARS-CoV-2 variants^15^ have considerably changed the clinical impact of SARS-CoV-2 infection on patients with cancer since March 2020.

To date, a significant gap in knowledge remains as to whether the positive effect of SARS-CoV-2 vaccination observed in the general population extends to patients with cancer treated with ICI. Recent evidence suggesting that ICI may precipitate subclinical cytokine release following SARS-CoV-2 vaccination^16^ strengthens the need to understand the relationship between COVID-19 vaccination and clinical outcomes.

With the aim of providing a contemporary description of COVID-19 morbidity and mortality in patients with cancer who were receiving ICIs at COVID-19 diagnosis and to assess the protective role of SARS-CoV-2 vaccination in this population, we developed this joint analysis of the OnCovid and European Society for Medical Oncology (ESMO) CoCARE registries.

## Methods

### Study design

OnCovid (NCT04393974) is a European registry study approved by the UK Health Research Authority (20/HRA/1608) collecting data from consecutive patients fulfilling the following inclusion criteria: (1) age ≥18 years; (2) Reverse transcription polymerase chain reaction (RT-PCR) confirmed diagnosis of SARS-CoV-2 infection; (3) history of solid or haematological malignancy either active or in remission at the time of COVID-19 diagnosis.

The ESMO-CoCARE is an observational prospective study, based on a longitudinal multicenter survey of patients with cancer with any solid or hematological malignancy who were diagnosed with COVID-19.

For both registries, data from consecutive, all-comer patients were collected using electronic case report forms designed with the Research Electronic Data Capture software (Vanderbilt University, Nashville, Tennessee, USA). Study details and procedures, patients’ eligibility, and clinical endpoints for both studies have already been extensively presented.^12–14 17–24^ A list of participating centers with eligible patients for the present analysis is provided as online supplemental table 1.

10.1136/jitc-2022-005732.supp1 Supplementary data

### Objectives and endpoints

The main objective of this analysis was to assess the protective role of SARS-CoV-2 vaccination in patients with cancer treated with a unique immunotherapy strategy, by comparing COVID-19 morbidity and mortality between unvaccinated and vaccinated patients.

In addition, we aimed to describe differences in COVID-19 severity and mortality depending on prior history of immune-related adverse events (irAEs) captured from ICI initiation until COVID-19 diagnosis.

Data of patients who received ICI within 3 months prior to COVID-19 diagnosis were merged from the OnCovid and ESMO CoCARE registries. Patients on chemotherapy-ICI and targeted therapy-ICI combinations were excluded from the analysis.

To reflect the temporal evolution of the pandemic, we first categorized patients according to date of COVID-19 diagnosis into prevaccination (from February 2020 to November 2020), alpha–delta (B.1.1.7–B.1.617.2) variants (from December 2020 to December 14, 2021), and omicron (B.1.1.529) variant (from December 15, 2021 to February 2022) pandemic phases as previously reported,^13^ and described COVID-19 mortality over time.

All-cause case fatality rate at 30 days (CFR_30_) was chosen as the main clinical endpoint, to differentiate early COVID-19-related mortality, from late, likely cancer-related deaths. As measures of COVID-19 morbidity, we evaluated the all-cause hospitalization and intensive care unit (ICU) admission rates, the rate of COVID-19 complications (at least one among acute respiratory failure, ARDS, kidney injury, secondary infections, sepsis, septic shock, acute cardiac injury, acute liver injury and others including thrombo-embolic events and other coagulopathies, autoimmune diseases, gastrointestinal reactions), the receipt of at least one COVID-19-oriented therapy (including antivirals, chloroquine-based treatment, antibiotics, corticosteroids, interleukin-6 inhibitors and others) (yes vs no), and supplemental oxygen therapy requirement (yes vs no).

Patients who received two doses of the BNT162b2, mRNA-1273, ChAdOx1-S, and CoronaVac vaccines prior to COVID-19, or in case of infection diagnosed at least 28 days after a single dose of the Ad.26.COV2.S vaccine, were defined as fully vaccinated. Patients who received one vaccination, without meeting the above-mentioned time criteria, were considered partially vaccinated, while patients who received a third dose of either the BNT162b2 or mRNA-1273 vaccine (or a second dose after the Ad.26.COV2.S vaccine) were considered boosted. Considering the limited sample size of vaccinated patients with breakthrough infections in the study population, and that the electronic case report form of the ESMO-CoCARE registry was not designed to collect information on booster doses, patients were grouped as unvaccinated (including partially vaccinated) and fully vaccinated (either double-dosed or boosted patients) for all the comparative analyses, while patients with unknown vaccination status were excluded.

For the irAEs analysis, we evaluated COVID-19 outcomes according to the experience of any grade (National Cancer Institute Common Toxicity Criteria for Adverse Events, V.5.0) treatment-related side effects with a putative immune-mediated mechanisms at any time prior to COVID-19. These were previously evaluated by clinicians at participating sites during routine consultations as clinically indicated, without predefined time points, and collected retrospectively by investigators.

Considering the recognized role of lymphopenia as prognostic biomarker in patients with COVID-19,^25^ we explored the association between the absolute lymphocyte count at COVID-19 (within 1 week of diagnosis) and the experience of prior irAEs in the subset of patients from the OnCovid registry. A detailed description of statistical analysis is provided as online supplemental methods.

## Results

### Study population

By the respective data lock dates of February 4, 2022 and May 17, 2022, the OnCovid and ESMO CoCARE included 3820 and 2310 patients. After the exclusion of ineligible patients, data from 178 (74.2%—OnCovid) and 62 (25.8%—ESMO CoCARE) patients diagnosed with COVID-19 between January 2020 and February 2022, who were receiving ICIs within 3 months prior to SARS-CoV-2 infection diagnosis, were merged.

**Figure 1:**
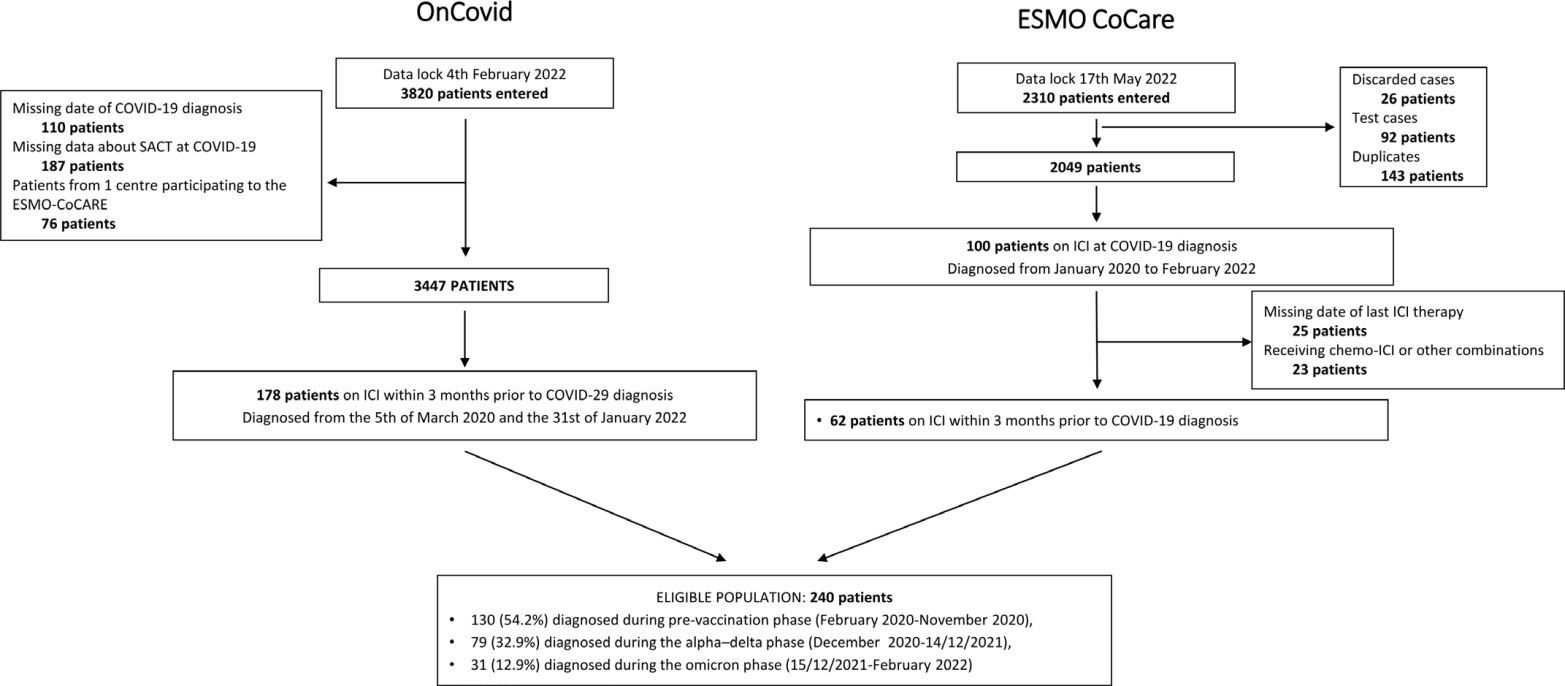
Study flow diagram. ESMO, European Society for Medical Oncology; ICI, immune checkpoint inhibitor; SACT, systemic anti-cancer therapies.

Figure 1 reports a detailed study flow diagram. The final study population consisted of 240 patients, of whom 130 (54.2%) were diagnosed with COVID-19 during the prevaccination phase, 79 (32.9%) during the alpha–delta phase, and 31 (12.9%) during the omicron phase, with reducing CFR_30_ over time: 25.8% (24/93 patients, 95% CI 16.5 to 38.4), 31.5% (17/54 patients, 95% CI 18.3 to 50.4), 3.6% (1/28 patients, 95% CI 0.09 to 19.8).

The most frequent primary tumor was lung cancer (47.1%), the majority of patients were male (67.5%), aged ≥65 years (62.1%), with at least one comorbidity (77.1%) and presented an active (76.7%), and advanced-stage (80.2%) tumor (table 1).

**Table 1:** Baseline patient characteristic and COVID-19 outcomes of the study population

	ICI population
	N=240 (%)
Country	
UK	47 (19.6)
Spain	77 (32.1)
Italy	90 (37.5)
Others	26 (10.8)
Sex	
Female	78 (32.5)
Male	162 (67.5)
Age	
<65 years	90 (37.5)
≥65 years	149 (62.1)
Missing	1 (0.4)
Comorbidities	
No	55 (22.9)
Yes	185 (77.1)
Primary tumor	
Lung	113 (47.1)
Melanoma	51 (21.2)
Others	76 (31.7)
Tumor stage	
Non-advanced	37 (15.6)
Advanced	190 (80.2)
Missing	10 (4.2)
Tumor status at COVID-19 diagnosis	
Remission/in-response	52 (21.7)
Active malignancy	184 (76.7)
Missing	4 (1.7)
SARS-CoV-2 vaccination status	
Unvaccinated	182 (75.8)
Fully vaccinated	42 (17.5)
Partially vaccinated	3 (1.3)
Unkown	13 (5.4)
COVID-19 outcomes	
	**N (rate, 95% CI)**
Oxygen therapy Missing	81 (**37.6**, 29.9 to 46.8) 25
COVID-19-specific therapy Missing	114 (**51.6**, 42.5 to 61.9) 19
Complications from COVID-19	73 (**30.4**, 23.8 to 38.2)
Hospitalization Missing	131 (**56.2**, 47.0 to 66.7) 7
ICU admission Missing	22 (**9.4**, 5.9 to 14.3) 7
30-days case fatality rate Missing	55 (**23.6**, 17.8 to 30.7) 7

COVID-19 outcomes' rates are provided in bold.

ICI, immune checkpoint inhibitor; ICU, intensive care unit.

The received ICI-based regimens were: 136 (56.7%) PD-1 inhibitors monotherapy, 54 (22.5%) PD-L1 inhibitors monotherapy, 20 (8.3%) others/experimental ICIs, 19 (7.9%) CTLA-4/PD-1 inhibitors combinations and 11 (4.6%) not specified chemotherapy-free ICI regimens.

Most patients were unvaccinated prior to COVID-19 (75.8%), 17.5% were fully vaccinated, 1.3% partially vaccinated, while vaccination status was unknown for 13 patients (5.4%). Among fully vaccinated patients, 17 from the OnCovid registry received a booster dose. Vaccination details for both the registries are summarized in online supplemental table 2.

The median observation period for the whole cohort was 91 days (IQR: 15.8–319.0) and the CFR_30_ was 23.6% (95% CI 17.8% to 30.7%). All COVID-19 outcomes for the whole cohort are summarized in table 1

### SARS-CoV-2 vaccination is associated with improvement in COVID-19 outcomes in ICI recipients

After the exclusion of 13 patients with unknown vaccination status, 227 patients were included in the SARS-CoV-2 vaccine analysis.

None of the baseline demographics and oncological characteristics were associated with SARS-CoV-2 vaccination status, with the exception of a higher proportion of patients with at least one comorbidity among unvaccinated patients (80.5% vs 64.3%, p=0.0230) (online supplemental table 3).

**Figure 2:**
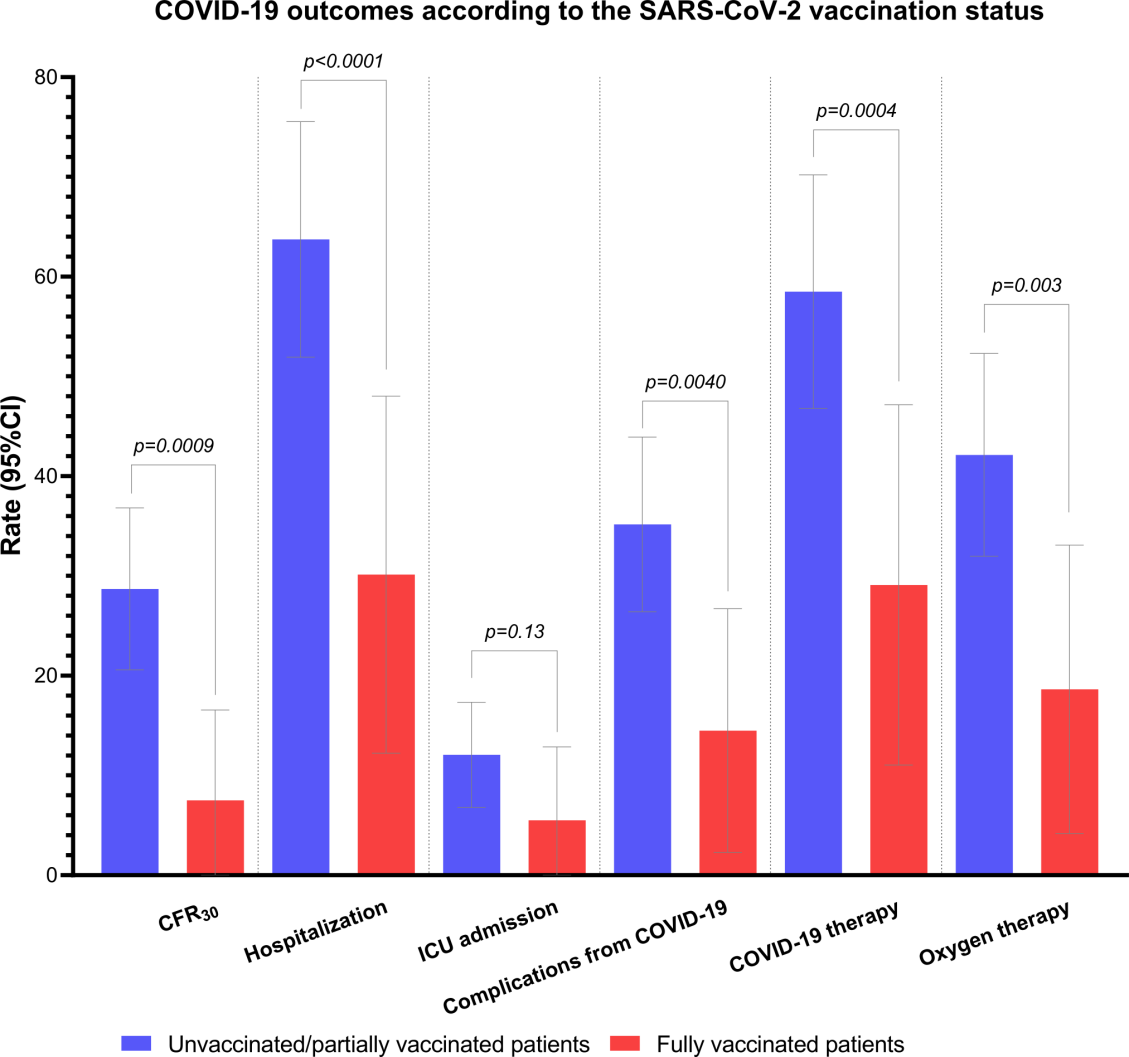
Histogram plot summarizing all COVID-19 outcomes according to the vaccination status. All rates with 95% CIs are available in online supplemental table 4. CFR30, 30-day case fatality rate; ICU, intensive care unit.

Univariable analysis revealed that fully vaccinated patients experienced decreased rates of death at 30 days (4.8% vs 28.1%, p=0.0009), hospitalization (27.5% vs 63.2%, p<0.0001), COVID-19 complications (11.9% vs 34.6%, p=0.0040), reduced need for COVID-19-specific therapy (26.3% vs 57.9%, p=0.0004) and oxygen therapy (15.8% vs 41.5%, p=0.0030) in comparison to unvaccinated/partially vaccinated patients. We found no significant difference in terms of ICU admission rates, despite arithmetically fewer vaccinated patients being admitted to ICU (4.8% vs 28.1%, p=0.14) (figure 2, online supplemental table 4).

**Figure 3:**
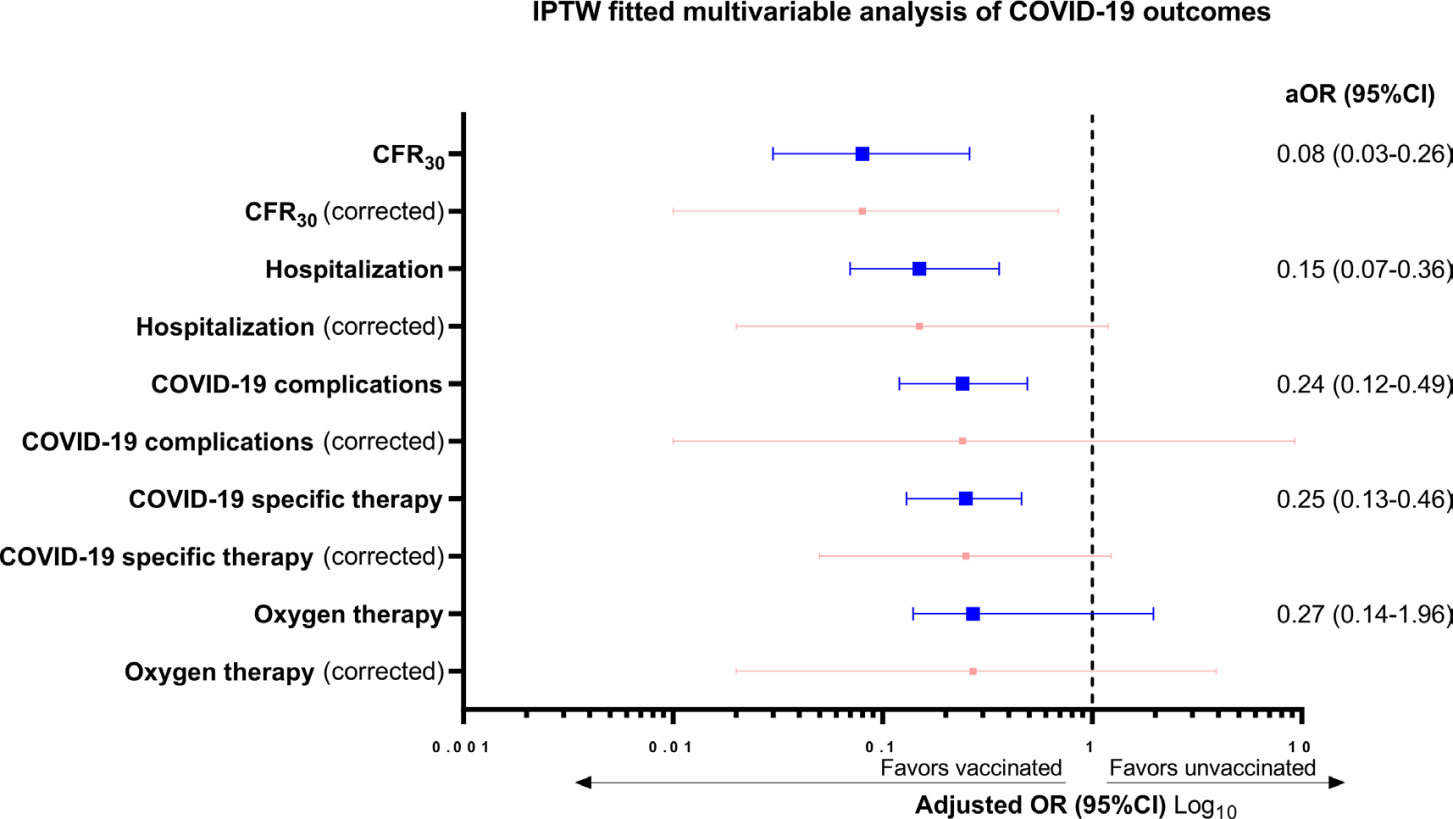
Summary of the inverse probability of treatment weighing (IPTW) fitted multivariable analyses for each COVID-19 outcomes according to the vaccination status prior to (blue) and after (red) the clustered-robust SE and 95% CI adjustments for the data source. Adjusting covariates for each COVID-19 outcome were country of origin, comorbidities, tumor status, and tumor stage at COVID-19. Full multivariable models are available in online supplemental table 6. aOR, adjusted OR; CFR30, 30-day case fatality rate.

Distribution of baseline patient characteristics prior to and after inverse probability of treatment weighting (IPTW) is reported in online supplemental table 5. Given the suboptimal balancing ability, country, comorbidities, tumor status and tumor stage were included in all IPTW-fitted multivariable logistic regression models for each COVID-19 outcome, which are reported in full as online supplemental table 6 and are summarized in the forest plot graph provided in figure 3. Compared with unvaccinated patients, full vaccination was associated with a decreased risk of death at 30 days (adjusted OR, aOR 0.08, 95% CI 0.03 to 0.26), of hospitalization (aOR 0.15, 95% CI 0.07 to 0.36), of COVID-19 complications (aOR 0.24, 95% CI 0.12 to 0.49) and of need for COVID-19-specific therapy (aOR 0.25, 95% CI 0.13 to 0.46). However, after clustered-robust correction for data source, the upper limit CI crosses one for all COVID-19 outcomes except for CFR_30_ (aOR 0.08, 95% CI 0.01 to 0.69).

### History of irAEs prior to COVID-19 is associated with decreased COVID-19 mortality in patients receiving ICI

Overall, 38 patients (15.8%) experienced any grade irAEs at any time prior to COVID-19, which are summarized in online supplemental table 7. The median time from occurrence of irAEs and COVID-19 diagnosis was 3.2 months (range 0.13–48.7, computed on data of 27 patients from the OnCovid registry).

The occurrence of irAEs was not associated with any of the baseline demographics and oncological characteristics, including the disease status (active vs remissive/in response) at COVID-19 (p=0.5339), with the exception of the primary tumor (p=0.0373) (online supplemental table 8).

**Figure 4:**
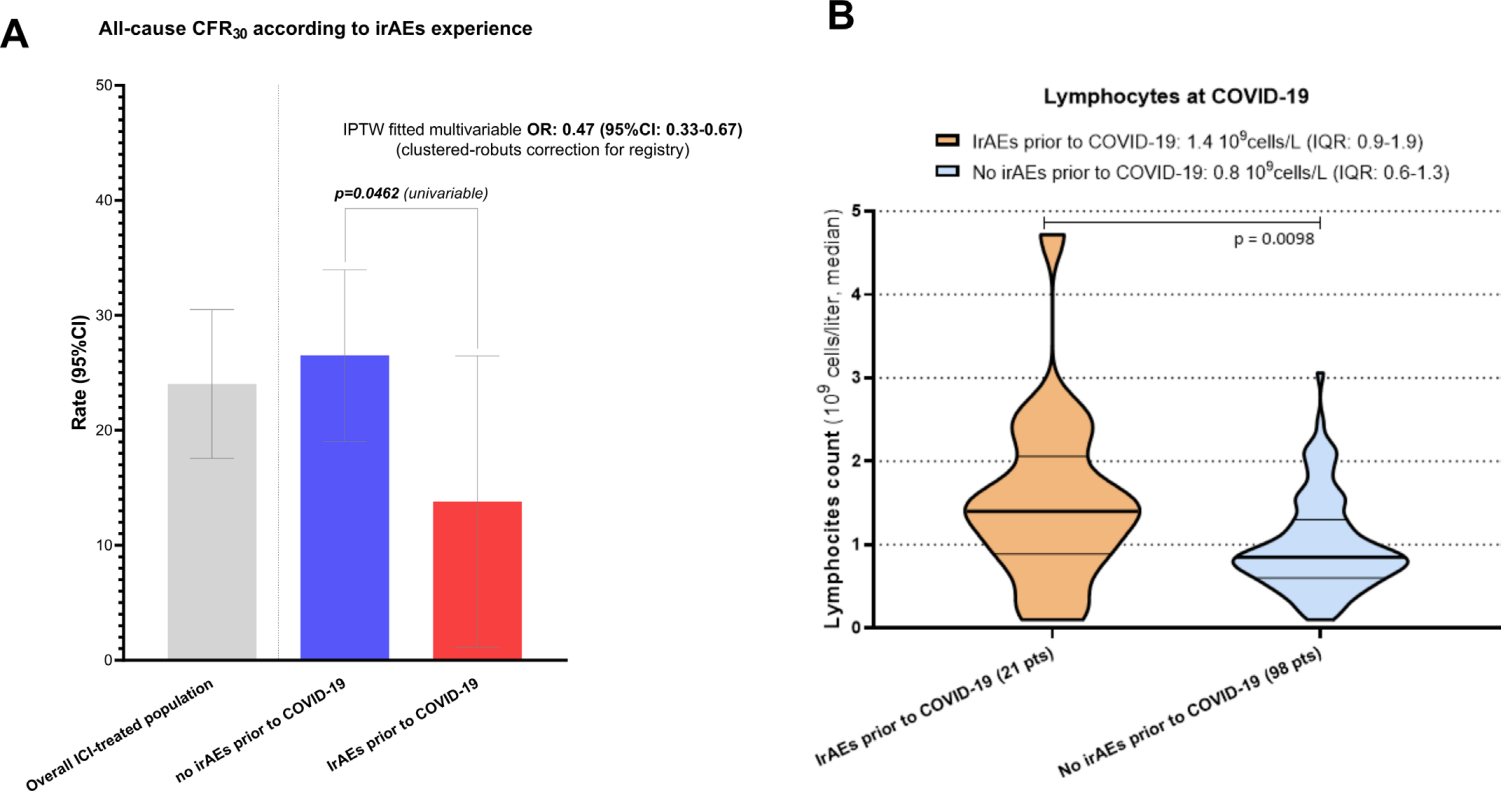
(A) Histogram plot summarizing the all-cause 30-day case fatality rate (CFR30) analysis according to the occurrence of any grade immune-related adverse events prior to COVID-19. Inverse probability of treatment weighing (IPTW) fitted adjusted OR for the risk of death at 30 days with clustered robust 95% CI correction for the data source is presented. All rates with 95% CI are available in online supplemental table 9. Adjusting covariates were country of origin, primary tumor, tumor stage at COVID-19 and vaccination status. Full multivariable model is available in online supplemental table 11. (B) Violin plot reporting the median absolute lymphocyte count at COVID-19 (within 1 week of diagnosis) according to the prior occurrence or any grade irAEs. IrAEs, immune-related adverse events.

Univariable analysis showed similar rates of hospitalization (51.3% vs 57.1%, p=0.5158), ICU admission (16.2% vs 8.1%, p=0.1252), COVID-19 complications (23.7% vs 31.7%, p=0.3265), COVID-19-specific therapy (45.7% vs 52.6%, p=0.4498) and oxygen requirement (39.3% vs 37.4%, p=0.8251) between patients who experienced and those who did not experience irAEs prior to COVID-19 (online supplemental table 9). However, the occurrence of irAEs was associated with a significantly decreased CFR_30_ (10.8% vs 26.0%, p=0.0462) (figure 4A).

Distribution of baseline characteristics distribution prior to and after the IPTW is reported in online supplemental table 10. Given the suboptimal balancing ability, country, tumor stage, primary tumor and vaccination status were included in the IPTW-fitted multivariable logistic regression model for COVID-19 mortality, which confirmed that patients who experienced any grade irAEs prior to COVID-19 had a decreased risk of death at 30 days (aOR 0.47, 95% CI 0.23 to 0.99). Clustered-robust correction for data source further strengthened this finding (aOR 0.47, 95% CI 0.33 to 0.67) (online supplemental table 11).

Lastly, in a subset of patients from the OnCovid cohort, we revealed that the median absolute lymphocyte count within 1 week of COVID-19 diagnosis was significantly higher among patients who experienced any grade irAEs prior to COVID-19 than in those who did not experience irAEs (1.4 vs 0.8 10^9^ cells/L, p=0.0098) (figure 4B).

## Discussion

Our study is the largest analysis on patients with cancer on ICIs diagnosed with COVID-19 to date. With the inclusion of patients diagnosed up until February 2022, it provides a more contemporary picture of COVID-19 outcomes in this specific population. Although merely descriptive due to the limited sample size of subgroups, the reducing CFR_30_ across the pandemic phases suggests a time-dependent improvement of COVID-19 mortality, especially during the more recent Omicron outbreak, as already reported for the OnCovid population.^13^

Even considering the time requirements for the delivery of immunization campaigns since the first SARS-CoV-2 vaccine approval,^26^ and that most of the included patients were diagnosed during the prevaccination phase, we consider 17.5% of full vaccination a relatively low rate, and a possible impact of vaccine hesitancy, as initially reported in early 2021,^27 28^ cannot be excluded.

Although preliminary evidence from clinical trials supports the safety and immunogenicity of SARS-CoV-2 vaccines in patients with cancer on active ICI-based treatments,^16 29 30^ this study demonstrates the efficacy of anti-COVID-19 vaccination in patients receiving ICI in routine clinical practice. The ~83% reduction in the CFR_30_ in fully vaccinated patients along with COVID-19-related morbidity is confirmed after adjustment for major prognostic confounders in IPTW-fitted models, a process made necessary by the inherent differences existing in study procedures and data collection modalities between the two registries.

The convergence of COVID-19 and ICI-toxicity in eliciting unopposed T-cell activation and downstream cytokine excess has been highlighted suggested as a hypothetical source of clinical risk to patients with cancer ever since the beginning of the pandemic.^5 31^ Contrary to initial concerns, we document an association between the occurrence of irAEs and reduced CFR_30_: a novel finding of potential interest in the development of COVID-19-specific therapeutics.

In our study, the protective role of irAEs of all grades on COVID-19-related mortality was independent of common clinicopathological features relating to cancer and COVID-19 prognosis, including SARS-CoV-2 vaccination status. It has been established that patients experiencing irAEs are those capable of mounting a more vigorous anticancer immune reconstitution, resulting in longer survival.^32^ Because T-cell exhaustion is not solely a hallmark of cancer progression but a mechanism of COVID-19 severity,^25 31^ we speculate whether history of prior irAE might be a surrogate of more functional T-cell immunity, leading to improved mortality from COVID-19 irrespective of vaccine status.

In keeping with this view, we found that the absolute lymphocyte count at COVID-19, was significantly higher among patients who experienced prior irAEs. It is well known that patients with severe COVID-19 show reduced counts of peripheral CD4+and CD8+ T cells^31^, and that reduced CD4+/CD8+T cells, B cells, NK cells, and absolute lymphocyte cell count levels are significantly associated with COVID-19 mortality in the general population.^25^ At the same time, the known mechanisms leading to irAEs involve expansion of intratumoral and peripheral T-cell receptor repertoires along with a mobilization of large numbers of T cells^33 34^ and, to a lesser extent, activation and exhaustion of CD21^low^ B cells.^35^ On the other hand, a decrease in the absolute lymphocyte count has been reported with severe ICI-associated myocarditis.^36^

While OnCovid and ESMO CoCARE registries lack information on T-cell phenotype at COVID-19 diagnosis, our findings are provocative in suggesting that prior irAE might represent a hallmark of protection from COVID-19 mortality through invigorated T-cell immunity. These findings deserve further mechanistic studies to fully elucidate the immunological links between irAEs and COVID-19 outcomes in patients with cancer.

Our study acknowledges several limitations, including lack of data regarding the smoking status and more detailed information regarding irAEs duration and management. Of note, previous irAEs and their putative immune-mediated mechanism were assessed at participating site in routine practice, without predefined time points. This might have impacted the quality of data with risks of underreporting, as the 16.7% and 3.1% rates of all grade and ≥G3 irAEs, respectively, are lower than those reported in interventional clinical trials with ICI-based regimens,^37^ but comparable to reports from clinical practice.^38^

In addition, inherent differences between the two registries significantly impacted the accuracy of the estimates from the vaccination analysis: information about booster doses only recently started to be collected for patients entered in the ESMO CoCARE registry and was not available for our analysis. Furthermore, for ~24% of vaccinated patients, the specific type of vaccine could not be reconstructed. While constituting an important limitation, this is unlikely to have affected our results, given recent evidence suggesting largely comparable efficacy of commonly available SARS-CoV-2 vaccines.^39^

Lastly, despite the inclusion of a significant proportion of more recently diagnosed patients, the lack of availability of viral genomic sequences across the pandemic phases did not allow us to make conclusive considerations about new SARS-CoV-2 variants, while the limited sample size of the ‘alpha–delta’ and ‘omicron’ phases subgroups prevented us from running adequately powered time-adjusted analyses.

Despite the mentioned limitations, our results collectively support the notion that ICI recipients are not especially vulnerable to COVID-19, with mortality rates that are in keeping with the general population with COVID-19 and cancer. In these patients, SARS-CoV-2 vaccination leads to significantly improved outcome from COVID-19, comparably with other oncological patient populations.^13 14 40^ Considering the continuously expanding indication for ICI therapy,^41^ our findings are of utmost importance to ensure effective utilization of this therapy during and beyond the SARS-CoV-2 global epidemic.

## Data Availability

Data are available on reasonable request. Individual, deidentified participant data and data dictionary may be made available at the request of investigators whose proposed use of the data has been approved by the OnCovid consortium and ESMO CoCARE steering committees following review of a methodologically sound research proposal.
