# Supplementary material for: Immune checkpoint inhibitor therapy and outcomes from SARS-CoV-2 infection in patients with cancer: a joint analysis of OnCovid and ESMO-CoCARE registries
Source: J Immunother Cancer. 2022 Nov 30;10(11):e005732. doi: 10.1136/jitc-2022-005732 (PMC9716413; doi:10.1136/jitc-2022-005732)
Supplement: Supplementary data [file jitc-2022-005732supp001.pdf]

Immune checkpoint inhibitor therapy and outcomes from SARS-CoV-2 infection in patients with cancer: a joint analysis of OnCovid and ESMO-CoCARE registries.

Cortellini A et al.

Contents

**Supplementary Methods**	**Page 2**
**Supplementary Table 1**	**Page 3**
**Supplementary Table 2**	**Page 4**
**Supplementary Table 3**	**Page 4**
**Supplementary Table 4**	**Page 5**
**Supplementary Table 5**	**Page 5**
**Supplementary Table 6**	**Page 6**
**Supplementary Table 7**	**Page 7**
**Supplementary Table 8**	**Page 7**
**Supplementary Table 9**	**Page 8**
**Supplementary Table 10**	**Page 8**
**Supplementary Table 11**	**Page 9**

Supplementary Methods

Statistical plan

Baseline characteristics were summarized as categorical variables and reported using descriptive statistics. We tested associations between categorical variables using the Fisher exact test and the Pearson χ2 test as appropriate. The Kurkas -Wallis test was used to compare ambsolute lymphocytes counts. COVID-19 outcomes were presented as crude rates with 95% confidence intervals (95%CI).

To optimize the unbalanced sample size of subgroups we performed dedicated Inverse Probability of Treatment Weighting (IPTW) procedures accounting for selected demographics and oncological characteristics for both the vaccination and irAEs analyses. The balancing ability of each IPTW was evaluated through the distribution of the unweighted and weighted selected variables with relevant p-values and standardised mean difference (SMD). Double adjustment for variables with a SMD >0.10 was adopted when exploring clinical outcomes between the weighed cohorts with multivariable analyses(1).

For the multivariable analyses propensity score-weighted logistic regression models were fitted for each COVID-19 outcome of interest, with results presented as adjusted odds ratios (aOR) and 95%CI.

To obtain a more powered IPTW we included variables with missing data by grouping them as reference term in case of a <3% of missingness and as an "unknown" category in case of a ≥3% of missingness.

The following covariates were merged from the registries and included in the IPTW procedures: country (United Kingdom vs Spain vs Italy vs others), sex (male vs female), age (≥65 vs <65 years), presence of at least one comorbidity (yes vs no), tumour status at COVID-19 (presence of active/progressive or stable disease vs remissive/in response disease), tumour stage (advanced vs non-advanced vs unknown) and primary tumour (clustered as lung vs melanoma/skin cancers vs others).

Vaccination status was included in the IPTW procedure for the irAEs analysis.

Acknowledging that the data-source consisted of 2 registries with different procedures and data collections modalities, all the results from multivariable analyses were corrected following a clustered-robust standard error and 95%CI correction according to the data source (OnCovid vs ESMO-CoCARE).

All P-values were 2-sided and confidence intervals set at the 95% level, with significance pre-defined to be at <0.05.

Analyses were performed using the R-studio software, R Core Team (2021). R: A language and environment for statistical computing. R Foundation for Statistical Computing, Vienna, Austria, and the MedCalc® Statistical Software version 20 (MedCalc Software Ltd, Ostend, Belgium; https://www.medcalc.org; 2021).

Reference

1. Nguyen, TL., Collins, G.S., Spence, J. et al. Double-adjustment in propensity score matching analysis: choosing a threshold for considering residual imbalance. BMC Med Res Methodol 17, 78 (2017). https://doi.org/10.1186/s12874-017-0338-0

Supplementary Table 1: Participating centers list with eligible patients from the OnCovid and ESMO-CoCARE registries.

Centre – OnCovid	Eligible patients	(%)

Vall d'Hebron University Hospital, Barcelona (Spain)	29	16.3%
ICO Girona (Spain)	12	6.7%
Ospedale Maggiore della Carità, Novara (Italy)	11	6.2%
Institut Gustave Roussy, Villejuif (France)	11	6.2%
Policlinico San Matteo, Pavia (Italy)	10	5.6%
Ospedale Papa Giovanni XXIII, Bergamo (Italy)	10	5.1%
IRCCS AOU San Martino, Genova (Italy)	10	5.1%
Chelsea and Westminster Hospital, London (UK)	9	5.1%
Barts Health NHS Trust, London (UK)	8	4.5%
Careggi University Hospital, Florence (Italy)	8	3.9%
IRCCS Humanitas Research Hospital, Rozzano - Milan (Italy)	8	3.9%
University of Bari 'Aldo Moro', Bari (Italy)	7	3.4%
Hospital Clinic, Barcelona (Spain)	6	3.4%
Imperial College London, London (UK)	5	2.8%
Guy's and St Thomas' NHS Foundation Trust, London (UK)	5	2.8%
ICO L'Hospitalet, L'Hospitalet de Llobregat, Barcelona (Spain)	4	2.2%
Istituto Europeo di Oncologia, Milano (Italy)	4	2.2%
Azienda Ospedaliera Spedali Civili, Brescia (Italy)	4	1.7%
Ospedali Riuniti di Ancona, Universitá Politecnica delle Marche (Italy)	4	1.7%
Università Campus Bio-Medico, Rome (Italy)	3	1.1%
ICO Badalona (Spain)	2	1.1%
Azienda Ospedaliera S Maria, Terni (Italy)	2	1.1%
Institut Jules Bordet, Brussels (Belgium)	1	0.6%
University of L'Aquila, L'Aquila (Italy)	2	0.6%
Santa Maria Goretti Hospital, Latina (Italy)	1	0.6%
Azienda Ospedaliera S. Andrea, Rome (Italy)	2	0.6%
**Total**	**178**	**100.0%**
**Centre – ESMO CoCARE**	**Eligible patients**	**(%)**
The Royal Marsden NHS Foundation Trust, London (UK)	20	32.3%
Hospital Universitario Infanta Leonor, Madrid (Spain)	10	16.1%
Hospital Universitario La Paz, Madrid (Spain)	7	11.3%
CHUV Lausanne (Switzerland)	5	8.1%
Hospital Universitario Ramón y Cajal, Madrid (Spain)	4	6.5%
Azienda Ospedaliera "SS Antonio e Biagio e C.Arrigo", Alessandria (Italy)	4	6.5%
H. Universitario Fundación Alcorcón, Madrid (Spain)	3	4.8%
Hospital Prof Doutor Fernando Fonseca, Llisbon (Portugal)	2	3.2%
4th Oncology Dept & Comprehensive Clinical Trials Center, Metropolitan Hospital Athens (Greece)	2	3.2%
401 General Military Hospital of Athens (Greece)	1	1.6%
Sechenov University Hospital, Moscow (Russia)	1	1.6%
Samsung Medical Center, Seul (South Korea)	1	1.6%
Asian Cancer Institute - Asian Hospital and Medical Center, Muntinlupa (Philippines)	1	1.6%
Fundeni Clinical Institute, Department of Medical Oncology, Bucharest (Romania)	1	1.6%
**Total**	**62**	**100.0%**

Supplementary Table 2: Vaccination details for patients with breakthrough infections from the OnCovid and ESMO CoCARE registries.

**Oncovid**			
	**Partially vaccinated N (%)**	**Double-dosed N (%)**	**Boosted N (%)**
**BNT162b2**	-	7 (43.7)	10 (58.8)
**mRNA-1273**	2 (100)	3 (18.7)	5 (29.4)
**Ad.26.COV2.S**	-	1 (6.2)	-
**Not specified**	-	5 (31.3)	2 (17.8)

**Total**	2	16	17

**CoCare**		
	**Partially vaccinated N (%)**	**Double dosed N (%)**
**BNT162b2**	-	2 (22.2)
**ChAdOx1-S**	-	4 (44.4)
**CoronaVac**	1 (100)	-
**Not specified**	-	3 (33.3)
**Total**	1	9

Supplementary Table 3: Baseline demographics and oncological characteristics according to the SARS-CoV-2 vaccination status. 13 patients with unknown vaccinations status have been excluded.

	**Unvaccinated/Partially**	**Fully Vaccinated**	**P value**
	**N = 185 (%)**	**N = 42 (%)**	
**Country**			
United Kingdom	34 (18.4)	11 (26.2)	0.1764
Spain	66 (35.7)	11 (26.2)	
Italy	61 (33.0)	18 (42.9)	
Others	24 (13.0)	2 (4.8)	
**Sex**			
Female	57 (30.8)	15 (35.7)	0.5385
Male	128 (69.2)	27 (64.3)	
**Age**			
<65 years	70 (37.8)	17 (40.5)	0.8548
≥65 years	114 (61.6)	25 (59.5)	
Missing	1 (0.5)	-	
**Comorbidities**			
No	36 (19.5)	15 (35.7)	0.0230
Yes	149 (80.5)	27 (64.3)	
**Primary Tumour**			
Lung	84 (45.4)	20 (47.6)	0.9481
Melanoma	39 (21.1)	9 (21.4)	
Others	62 (33.5)	13 (31.0)	
**Tumour stage**			
Non-advanced	25 (13.5)	8 (19.0)	0.0866
Advanced	152 (82.2)	29 (69.0)	
Missing	8 (4.3)	5 (11.9)	
**Status at COVID-19 diagnosis**			
Remission/in-response	40 (21.6)	11 (26.2)	0.1962
Active malignancy	143 (77.3)	29 (69.0)	
Missing	2 (1.1)	2 (4.8)	
**IrAEs prior to COVID-19**			
No	155 (83.8)	34 (81.0)	0.6580
Yes	30 (16.2)	8 (19.0)	

Supplementary Table 4: Summary and univariable analysis of COVID-19 outcomes among vaccinated and unvaccinated patients.

	**Unvaccinated/Partially (N=185)**	**Fully Vaccinated (N=42)**	**p-value**
	**N (Rate, 95%CI)**	**N (Rate, 95%CI)**	
**Oxygen therapy**<br>Missing	69 (**41.5**, 32.3-52.6)<br>19	6 (**15.8**, 5.8-34.3)<br>4	0.0030
**COVID-19 specific therapy**<br>Missing	99 (**57.9**, 47.1-70.5)<br>14	10 (**26.3**, 12.6-48.4)<br>4	0.0004

**Complications from COVID-19**	64 (**34.6**, 26.7-44.2)	5 (**11.9**, 3.8-27.8)	0.0040
**Hospitalization**<br>Missing	115 (**63.2**, 52.2-75.8)<br>3	11 (**27.5**, 13.7-49.2)<br>2	<0.0001
**ICU admission**<br>Missing	21 (**11.5**, 7.1-17.6)<br>3	1 (**2.5,** 0.1-13.9)<br>2	0.1387
**30-days case fatality rate**<br>Missing	51 (**28.1**, 20.9-37.1)<br>4	2 (**4.8**, 0.1-17.6)<br>1	0.0009

Supplementary Table 5: Distribution of baseline characteristics before and after the IPTW procedure between unvaccinated and vaccinated patients included in the vaccination analysis. Variability of included characteristics is estimated through the standardized mean difference (SMD).

	Unvaccinated (%)	Fully vaccinated (%)	P value	SMD	Unvaccinated Weighted (%)	Fully vaccinated Weighted (%)	P value Weighted	SMD Weighted
**Country**								
United Kingdom	18.4	26.2	0.17	0.40	19.4	16.1	0.86	0.16
Spain	35.7	26.2			34.5	37.8		
Italy	33.0	42.9			34.4	38.0		
Others	13.0	4.8			11.7	8.1		
**Sex**								
Male	69.2	64.3	0.66	0.10	69.5	68.4	0.90	0.02
**Age**								
≥65 years	61.6	59.5	0.93	0.04	62.0	59.3	0.76	0.05
**Comorbidities**								
Yes	80.5	64.3	0.04	0.37	80.3	66.3	0.08	0.32
**Status at COVID-19**								
Active malignancy	77.3	69.0	0.35	0.19	77.2	65.6	0.16	0.25
**Tumour stage**								
Non-advanced	13.5	19.5	0.20	0.29	13.6	21.9	0.31	0.26
Advanced	82.2	70.7			82.2	71.2		
Unknown	4.3	9.8			4.2	6.8		
**Primary tumours**								
Lung	45.4	47.6	0.94	0.06	45.6	44.1	0.98	0.03
Melanoma	21.1	21.4			20.7	21.8		
Others	33.5	31.0			33.7	34.1		

Supplementary Table 6: Full fitted multivariable logistic regression models after the Inverse Probability of Treatment Weighting (IPTW) procedure for each COVID-19 related outcome comparing all vaccinated patients and unvaccinated patients. Standard errors and adjusted OR with 95%CIs before and after the clustered-robust adjustment for the data source (OnCovid vs ESMO CoCARE) are presented. UK: United Kingdom; Unk: unknown; aOR: adjusted odds ratio; CI: confidence intervals; St. Err: standard error.

30-days Case Fatality Rate	aOR	95%CI	St. Err	95%CI cluster-corrected	St. Err cluster-corrected
**Vaccination status:** Full vaccination vs unvaccinated	0.08	0.03-0.26	0.55	0.01-0.69	1.06

**Country:** Spain vs UK	1.11	0.38-3.24	0.54	0.40-3.04	0.51
**Country:** Italy vs UK	0.81	0.27-2.35	0.54	0.14-4.65	0.89
**Country:** Others vs UK	0.80	0.16-3.88	0.80	0.28-2.23	0.52
**Comorbidities:** Yes vs No	1.73	0.62-4.88	0.52	0.90-3.33	0.33
**Tumour status:** Active malignancy vs Remission/response	4.97	1.34-18.43	0.66	2.89-8.53	0.27
**Tumour stage at COVID-19:** Advanced vs Non-advanced	1.71	0.43-6.85	0.70	0.99-2.97	0.28
**Tumour stage at COVID-19:** Unk vs Non-advanced	0.33	0.02-6.08	1.48	0.15-0.72	0.39
**Hospitalization** (all causes)	**aOR**	**95%CI**	**St. Err**	**95%CI cluster-corrected**	**St. Err cluster-corrected**
**Vaccination status:** Full vaccination vs unvaccinated	0.15	0.07-0.36	0.42	0.02-1.19	1.02
**Country:** Spain vs UK	5.19	1.85-14.6	0.52	4.79-5.63	0.04
**Country:** Italy vs UK	0.25	0.09-0.70	0.52	0.03-1.73	0.98
**Country:** Others vs UK	0.58	0.12-2.90	0.82	0.24-1.39	0.44
**Comorbidities:** Yes vs No	2.34	0.89-6.18	0.49	0.92-5.94	0.47
**Tumour status:** Active malignancy vs Remission/response	6.78	2.33-19.79	0.54	4.02-11.4	0.26
**Tumour stage at COVID-19:** Advanced vs Non-advanced	1.01	0.33-3.02	0.56	0.92-1.07	0.03
**Tumour stage at COVID-19:** Unk vs Non-advanced	0.22	0.03-1.31	0.90	0.02-3.80	1.45
**COVID-19 complications**	**aOR**	**95%CI**	**St. Err**	**95%CI cluster-corrected**	**St. Err cluster-corrected**
**Vaccination status:** Full vaccination vs unvaccinated	0.24	0.12-0.49	0.35	0.01-9.25	1.85
**Country:** Spain vs UK	1.23	0.51-2.97	0.44	0.69-2.21	0.29
**Country:** Italy vs UK	0.28	0.10-0.78	0.51	0.12-0.64	0.42
**Country:** Others vs UK	1.23	0.35-4.34	0.64	0.28-5.31	0.74
**Comorbidities:** Yes vs No	0.92	0.41-2.09	0.41	0.19-4.38	0.79
**Tumour status:** Active malignancy vs Remission/response	1.43	0.62-3.34	0.43	0.85-2.38	0.26
**Tumour stage at COVID-19:** Advanced vs Non-advanced	1.35	0.48-3.81	0.53	0.35-5.17	0.68
**Tumour stage at COVID-19:** Unk vs Non-advanced	0.41	0.05-2.93	1.00	0.05-2.87	0.99
**COVID-19 specific therapy**	**aOR**	**95%CI**	**St. Err**	**95%CI cluster-corrected**	**St. Err cluster-corrected**
**Vaccination status:** Full vaccination vs unvaccinated	0.25	0.13-0.46	0.31	0.05-1.23	0.81
**Country:** Spain vs UK	4.24	1.62-11.07	0.49	1.17-15.28	0.65
**Country:** Italy vs UK	2.38	0.88-6.41	0.50	1.69-3.36	0.17
**Country:** Others vs UK	3.94	1.02-15.10	0.68	2.23-6.96	0.29
**Comorbidities:** Yes vs No	1.22	0.55-2.72	0.41	0.36-4.11	0.62
**Tumour status:** Active malignancy vs Remission/response	0.91	0.43-1.94	0.38	0.87-0.96	0.02
**Tumour stage at COVID-19:** Advanced vs Non-advanced	1.02	0.40-2.59	0.47	0.74-1.39	0.15
**Tumour stage at COVID-19:** Unk vs Non-advanced	1.79	0.38-8.40	0.79	0.12-26.36	1.37
**Oxygen therapy**	**aOR**	**95%CI**	**St. Err**	**95%CI cluster-corrected**	**St. Err cluster-corrected**
**Vaccination status:** Full vaccination vs unvaccinated	0.27	0.14-1.96	0.34	0.02-3.90	1.34
**Country:** Spain vs UK	2.54	0.99-6.48	0.47	1.84-3.49	0.16
**Country:** Italy vs UK	0.63	0.22-1.75	0.52	0.55-0.72	0.06
**Country:** Others vs UK	1.28	0.33-4.91	0.68	0.20-8.37	0.95
**Comorbidities:** Yes vs No	1.14	0.48-2.66	0.43	0.11-11.72	1.19
**Tumour status:** Active malignancy vs Remission/response	1.23	0.55-2.73	0.41	1.12-1.35	0.04
**Tumour stage at COVID-19:** Advanced vs Non-advanced	0.81	0.31-2.13	0.49	0.31-2.15	0.49
**Tumour stage at COVID-19:** Unk vs Non-advanced	0.11	0.01-1.34	1.25	0.02-0.78	0.98

Supplementary Table 7: Summary of the irAEs experienced prior to COVID-19 among the OnCovid and ESMO CoCARE cohorts. National Cancer Institute Common Toxicity Criteria for Adverse Events, version 5.0 were used for irAEs grading.

**30 patients from the OnCovid registry**	**8 patients from the CoCare registry**

Cortellini A, *et al. J Immunother Cancer* 2022; 10:e005732. doi: 10.1136/jitc-2022-005732

• Grade 3 colitis, n=1<br>• Grade 2 colitis, n=1<br>• Grade 2 pneumonitis, n=4<br>• Grade 2 skin reactions, n=3<br>• Grade 1 skin reactions, n= 2<br>• Grade 1 fatigue, n=1<br>• Grade 3 hepatitis, n=2<br>• Grade 2 hepatitis, n=2<br>• Grade 3 thyroiditis, n=1<br>• Grade 2 thyroiditis, n=5<br>• Grade 1 thyroiditis, n=2<br>• Grade 1 arthritis, n=1<br>• Grade 1 neuro-muscular reactions, n=2<br>• Grade 1 other reactions, n=3	• Grade 3 pneumonitis, n=1<br>• Grade 3 myocarditis, n=1<br>• Grade 3 hypothyroidism, n=1<br>• Grade 3 colitis, pneumonitis, hepatitis, thyroiditis, n=1<br>• Grade 2 myocarditis, hypothyroidism, n=1<br>• Grade 2 psoriasis, n=1<br>• Grade 1 thyroiditis, n=1

Supplementary Table 8: Baseline demographics and oncological characteristics according to the experience of immune-related adverse events (irAEs) prior to COVID-19 diagnosis.

	**No irAE**	**irAEs**	**P value**
	**N = 202 (%)**	**N = 38 (%)**	
**Country**			
United Kingdom	41 (20.3)	6 (15.8)	0.8947
Spain	65 (32.2)	12 (31.6)	
Italy	75 (37.1)	15 (39.5)	
Others	21 (10.4)	5 (13.2)	
**Sex**			
Female	66 (32.7)	12 (31.6)	0.8951
Male	136 (67.3)	26 (68.4)	
**Age**			
<65 years	76 (37.6)	14 (36.8)	0.9041
≥65 years	125 (61.9)	24 (63.2)	
Missing	1 (0.5)	-	
**Comorbidities**			
No	46 (22.8)	9 (23.7)	0.9025
Yes	156 (77.2)	29 (76.3)	
**Primary Tumour**			
Lung	99 (49.0)	14 (36.8)	0.0373
Melanoma	37 (18.3)	14 (36.8)	
Others	66 (32.7)	10 (26.3)	
**Tumour stage**			
Non-advanced	31 (15.6)	6 (15.8)	0.4645
Advanced	161 (80.9)	29 (76.3)	
Missing	7 (3.5)	3 (7.9)	
**Status at COVID-19 diagnosis**			
Remission/in-response	42 (20.8)	10 (26.3)	0.5339
Active malignancy	156 (77.2)	28 (73.7)	
Missing	4 (2.0)	-	
**SARS-CoV-2 vaccination status**			
Unvaccinated	153 (75.7)	29 (76.3)	0.6736
Fully vaccinated	34 (16.8)	8 (21.1)	
Partially vaccinated	3 (1.5)	-	
Unkown	12 (5.9)	1 (2.6)	

Supplementary Table 9: Summary and univariable analysis of COVID-19 outcomes according to the experience of any grade irAEs prior to COVID-19 diagnosis. irAEs: immune-related adverse events.

	**No IrAEs**	**IrAEs**	**p-value**

	**(N=202)**	**(N=38)**	
	**N (Rate, 95%CI)**	**N (Rate, 95%CI)**	
**Oxygen therapy**<br>Missing	68 (**37.4**, 29.0.6-43.4)<br>20	13 (**39.3**, 20.9-67.3)<br>5	0.8251
**COVID-19 specific therapy**<br>Missing	98 (**52.6**, 42.7-64.2)<br>16	16 (**45.7**, 26.1-74.2)<br>3	0.4498
**Complications from COVID-19**	64 (**31.7**, 24.4-40.5)	9 (**23.7**, 10.8-44.9)	0.3265
**Hospitalization**<br>Missing	112 (**57.1**, 47.1-68.7)<br>6	19 (**51.3**, 30.9-80.2)<br>1	0.5158
**ICU admission**<br>Missing	16 (**8.1**, 4.6-13.2)<br>6	6 (**16.2,** 5.9-35.3)<br>1	0.1252
**30-days case fatality rate**<br>Missing	51 (**26.0**, 19.3-34.2)<br>6	4 (**10.8**, 2.9-27.7)<br>1	0.0462

Supplementary Table 10: Distribution of baseline characteristics before and after the IPTW procedure between patients who experience and did not experience any grade irAEs prior to COVID-19 diagnosis. Variability of included characteristics is estimated through the standardized mean difference (SMD). irAEs: immune-related adverse events.

	**No irAEs (%)**	**IrAEs (%)**	**P value**	**SMD**	**No irAEs Weighted (%)**	**IrAEs Weighted (%)**	**P value Weighted**	**SMD Weighted**
**Country**								
United Kingdom	20.3	15.8	0.89	0.15	19.8	21.7	0.94	0.11
Spain	32.6	31.6			32.1	34.0		
Italy	37.1	39.5			37.3	32.0		
Others	10.4	13.2			10.8	12.3		
**Sex**								
Male	67.3	68.4	1.0	0.03	67.4	69.6	0.79	0.04
**Age**								
≥65 years	61.9	63.2	1.0	0.03	61.8	60.0	0.84	0.03
**Comorbidities**								
Yes	77.2	76.3	1.0	0.02	76.9	78.8	0.80	0.04
**Status at COVID-19**								
Active malignancy	77.2	73.7	0.79	0.08	76.6	73.8	0.72	0.06
**Tumour stage**								
Non-advanced	15.4	15.8	0.67	0.14	15.9	14.4	0.58	0.18
Advanced	80.1	76.3			79.5	76.2		
Unknown	4.5	7.9			4.6	9.4		
**Primary tumours**								
Lung	49.0	36.8	0.03	0.42	48.4	39.5	0.34	0.27
Melanoma	18.3	36.8			19.0	30.5		
Others	32.7	26.3			32.6	30.0		
**Vaccination status**								
Unvaccinated	77.2	76.3	0.61	0.19	76.8	77.7	0.71	0.15
Fully vaccinated	16.8	21.1			17.6	19.7		
Unknown	5.9	2.6			5.6	2.5		

Supplementary Table 11: Full fitted multivariable logistic regression model after the Inverse Probability of Treatment Weighting (IPTW) procedure for COVID-19 mortality comparing all patients who experienced and those who did not experience any grade irAEs prior to COVID-19. Standard errors and adjusted OR with 95%CIs before and after the clustered-robust adjustment for the data source (OnCovid vs ESMO CoCARE) are

presented. UK: United Kingdom; Unk: unknown; aOR: adjusted odds ratio; CI: confidence intervals; St. Err: standard error; irAEs: immune-related adverse events.

30-days Case Fatality Rate	aOR	95%CI	St. Err	95%CI cluster-corrected	St. Err cluster-corrected
**IrAEs prior to COVID-19:** Yes vs No	0.47	0.23-0.99	0.37	0.33-0.67	0.17
**Country:** Spain vs UK	0.77	0.26-2.26	0.54	0.30-1.97	0.47
**Country:** Italy vs UK	0.87	0.29-2.58	0.55	0.78-0.96	0.05
**Country:** Others vs UK	0.36	0.07-1.78	0.81	0.22-0.60	0.25
**Primary tumour:** Melanoma vs Lung	0.51	0.20-1.32	0.48	0.21-1.22	0.43
**Primary tumour:** Others vs Lung	0.36	0.15-0.88	0.44	0.16-0.82	0.41
**Tumour stage at COVID-19:** Advanced vs Non-advanced	3.97	0.94-16.6	0.73	0.81-19.2	0.80
**Tumour stage at COVID-19:** Unk vs Non-advanced	7.59	1.09-52.5	0.98	1.52-37.8	0.81
**Vaccination status:** Fully vaccinated vs unvaccinated/partially	0.07	0.01-0.45	0.95	0.03-0.17	0.44
**Vaccination status:** Unknown vs unvaccinated/partially	0.42	0.04-4.01	1.15	0.24-0.73	0.42

Cortellini A, *et al. J Immunother Cancer* 2022; 10:e005732. doi: 10.1136/jitc-2022-005732
